# Management of prostate cancer in older men: recommendations of a working group of the International Society of Geriatric Oncology

**DOI:** 10.1111/j.1464-410X.2010.09334.x

**Published:** 2010-08

**Authors:** Jean-Pierre Droz, Lodovico Balducci, Michel Bolla, Mark Emberton, John M Fitzpatrick, Steven Joniau, Michael W Kattan, Silvio Monfardini, Judd W Moul, Arash Naeim, Hendrik van Poppel, Fred Saad, Cora N Sternberg

**Affiliations:** 1 Department of Medical Oncology, Claude-Bernard-Lyon-1 University and Centre Léon-BérardLyon; 2 H. Lee Moffitt Cancer Center and Research InstituteTampa, FL; 3 Department of Radiation Therapy, Albert Michallon HospitalGrenoble, France; 4 Institute of Urology and Nephrology, University College LondonUK; 5 Mater Misericordiae University Hospital and University College DublinDublin, Ireland; 6 University Hospitals Leuven, Department of UrologyLeuven, Belgium; 7 Cleveland Clinic Lerner College of Medicine of Case Western Reserve University, Department of Quantitative Health Sciences, Cleveland ClinicCleveland, OH; 8 Geriatric Oncology Program Istituto Oncologico, Istituto PalazzoloMilano; 9 Duke University Medical Center, Division of Urologic SurgeryDurham, NC; 10 Division of Hematology-Oncology and Geriatrics, David Geffen School of Medicine, University of CaliforniaLos Angeles, CA, USA; 12 Chief, Department of Medical Oncology, San Camillo Forlanini HospitalRome, Italy; 11 Uro-Oncology Clinic, Centre Hospitalier de l’Université de Montreal, Hospital Notre-DameMontreal, Quebec, Canada

**Keywords:** elderly, guidelines, localized disease, metastatic, prostate cancer

## Abstract

Prostate cancer is the most prevalent cancer in men and predominantly affects older men (aged ≥70 years). The median age at diagnosis is 68 years; overall, two-thirds of prostate cancer-related deaths occur in men aged ≥75 years. With the exponential ageing of the population and the increasing life-expectancy in developed countries, the burden of prostate cancer is expected to increase dramatically in the future. To date, no specific guidelines on the management of prostate cancer in older men have been published. The International Society of Geriatric Oncology (SIOG) conducted a systematic bibliographic search based on screening, diagnostic procedures and treatment options for localized and advanced prostate cancer, to develop a proposal for recommendations that should provide the highest standard of care for older men with prostate cancer. The consensus of the SIOG Prostate Cancer Task Force is that older men with prostate cancer should be managed according to their individual health status, which is mainly driven by the severity of associated comorbid conditions, and not according to chronological age. Existing international recommendations (European Association of Urology, National Comprehensive Cancer Network, and American Urological Association) are the backbone for localized and advanced prostate cancer treatment, but need to be adapted to patient health status. Based on a rapid and simple evaluation, patients can be classified into four different groups: 1, ‘Healthy’ patients (controlled comorbidity, fully independent in daily living activities, no malnutrition) should receive the same treatment as younger patients; 2, ‘Vulnerable’ patients (reversible impairment) should receive standard treatment after medical intervention; 3, ‘Frail’ patients (irreversible impairment) should receive adapted treatment; 4, Patients who are ‘too sick’ with ‘terminal illness’ should receive only symptomatic palliative treatment.

## INTRODUCTION

Prostate cancer is the most frequently diagnosed male cancer in both the USA [1] and Europe [2]. It represents the second most common cause of cancer-related death in the USA [1] and the third cause in Europe [2]. Prostate cancer is predominantly a disease of older men (i.e. ≥70 years). According to the Surveillance, Epidemiology, and End Results database of the USA National Cancer Institute, the median age at diagnosis is 68 years and 71.2% of deaths due to prostate cancer occur in men aged ≥75 years [3]. With the exponential ageing of the population and the increasing life-expectancy, especially in developed countries, the burden due to prostate cancer is expected to increase dramatically in the future.

Existing guidelines for the management of patients with prostate cancer [4–6] do not make specific treatment recommendations for older men. Although advanced age alone should not preclude effective treatment for prostate cancer, it is necessary to assess the risks and benefits of treatment in each patient to avoid interventions that might decrease health-related quality of life (HRQL) without prolonging survival. Using a systematic review of available literature, the International Society of Geriatric Oncology (SIOG) developed recommendations for the assessment and treatment of older men with prostate cancer. The full version of these recommendations was published recently [7]. The aim of this review is to provide the urologist with a short summary of evidence-based recommendations, including specific decision algorithms, for managing older men with localized or advanced prostate cancer.

## EVALUATION OF LIFE-EXPECTANCY AND HEALTH STATUS

**FIG. 1:**
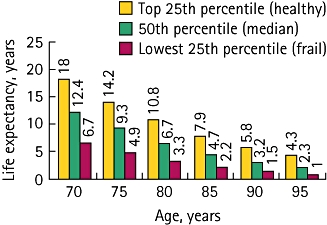
Life-expectancy in older men; there is a large variability reflecting variability in health status. Reprinted from [8], copyright (2001), with permission from the American Medical Association.

Life-expectancy is a major determinant of the potential for benefit from therapy beyond palliative care, yet it varies substantially between individuals within a given age group. Life-expectancy estimates apply to a population and represent a useful tool for public healthcare, but are not valid for a given individual. For example, 75-year-old men are expected to live for a further 8.3 years (median), but ≈25% (the upper quartile; likely to represent healthy individuals) will live for at least 14.2 years, whereas another 25% (the lower quartile; likely to represent frail individuals with significant comorbid conditions) will live for <4.9 years [8] (Fig. 1). Thus, although it is not possible to calculate the exact chance of survival for an individual, variables such as the number and severity of comorbidities and the extent of functional impairment can be used to predict the chance of surviving within an age group. Hence, it has been shown by Tewari *et al*. [9] that comorbidity evaluated by the Charlson index was the strongest predictor of death from other than prostate cancer in men with localized prostate cancer treated with radical prostatectomy (RP). Age was also a significant predictor of outcome, although to a lesser extent than comorbidity. It was also reported by Rockwood *et al*. [10] that senior adult patients who are dependent in daily living activities have a shorter survival.

Health status influences patient survival and might affect the ability to tolerate treatment-related side-effects. A previous SIOG Working Group concluded that screening for geriatric problems, using tools such as the Instrumental Activities of Daily Living (IADL), the Activities of Daily Living (ADL), the geriatric depression scale, and Folstein’s mini-mental status, was not sufficient for detecting age-related factors that would affect treatment outcomes in senior adults [11]. This screening stage should be followed by a more complete comprehensive geriatric assessment (CGA) which assesses various biological and clinical correlates of ageing on an individual basis, and includes diagnostic procedures, specific treatment plans, and geriatric intervention [7]. Several randomized controlled trials and meta-analyses have confirmed the benefit of CGA on survival, HRQL, institutionalization rates, and many other outcomes [11], and although few studies have specifically examined the benefit of CGA in geriatric oncology, the available evidence generally supports the effectiveness of such an approach.

## DEFINING PATIENT SUBGROUPS BY HEALTH STATUS

The SIOG Prostate Cancer Working Group reviewed recent literature and opinion on prognostic factors that might affect health status, overall survival, and prostate cancer-specific survival [7]. The most important factors to consider for the evaluation of health status in older men with prostate cancer were comorbidities, dependence status, and nutritional status.

### Comorbidities

Comorbidity is a major predictor of non-prostate cancer survival [9]. The Cumulative Illness Score Rating-Geriatrics (CISR-G) was judged to be the best available tool for assessing the risk for death unrelated to prostate cancer [12,13]. In contrast to the Charlson index, which considers only potentially lethal comorbid conditions, the CISR-G also rates nonlethal conditions according to their severity and level of control with treatment, as: Grade 0, no problem; Grade 1, current mild problem or past significant problem; Grade 2, moderate disability or morbidity, requires first-line therapy; Grade 3, severe/constant significant disability/uncontrollable chronic problem; and Grade 4, extremely severe/immediate treatment required/end-organ failure/severe impairment in function.

### Dependence status

There is evidence that the level of dependence in daily living activities influences survival in senior adult patients [10]. Dependence can be easily evaluated using the ADL [14] and IADL [15] scales.

The ADL scale rates a patient’s ability to accomplish basic activities of daily living (bathing, dressing, toileting, transferring, continence, and feeding). One ADL impairment is considered abnormal in older men with prostate cancer, with the exception of incontinence.

The IADL scale rates activities that require a higher level of cognition and judgement. Four items apply to men with prostate cancer: ability to manage money, to manage medications, to use transportation, and to use the telephone. One IADL impairment is considered abnormal in older men with prostate cancer.

### Nutritional status

Malnutrition has also been shown to be associated with an increased mortality rate in senior adult patients [16]. Nutritional status can be estimated simply by the variation of weight during the previous 3 months: Good nutritional status, <5% of weight loss; risk of malnutrition, weight loss 5-10%; severe malnutrition, weight loss >10%.

## EXPERT PANEL RECOMMENDATIONS

The SIOG Prostate Cancer Working Group recommends that the decision-making process for treating older men with prostate cancer should be based on a systematic evaluation of comorbidities (CISR-G scale), dependence status (IADL and ADL scales), and nutritional status (weight loss estimation). In cases of vulnerability and frailty, additional geriatric interventions including a CGA might be needed.

These tools enable older men with prostate cancer to be classified into one of four health status categories [17]:

1. *‘Healthy’ or ‘fit’*: the patient has no serious comorbidity (CISR-G Grade 0, 1 or 2), is functionally independent (no dependence in IADL and ADL), and has no malnutrition. The health status of these patients is considered to be sufficient to tolerate any form of standard cancer treatment.
2. *‘Vulnerable’*: the patient is dependent in one or more IADL (but no dependence in ADL); or presents with one comorbid uncontrolled condition (CISR-G Grade 3); or is at risk of malnutrition (weight loss 5–10% within the last 3 months). Geriatric problems in this group should be reversible through geriatric intervention. These patients might benefit from additional geriatric intervention, and they can receive standard cancer treatment after resolution of the geriatric problems.
3. *‘Frail’*: the patient is dependent in one or more ADL; or presents with two or more uncontrolled comorbid conditions (i.e. at least two comorbidities CISR-G Grade 3 or one comorbidity CISR-G Grade 4); or has severe malnutrition (weight loss >10% within the last 3 months). Patients in this group should benefit from geriatric intervention and can be given specifically adapted cancer treatment.
4. ‘T*oo sick’*: the patient has a very poor health status resulting from a combination of different impairments; likely to be suitable for palliative end-of-life treatment only.

**FIG. 2:**
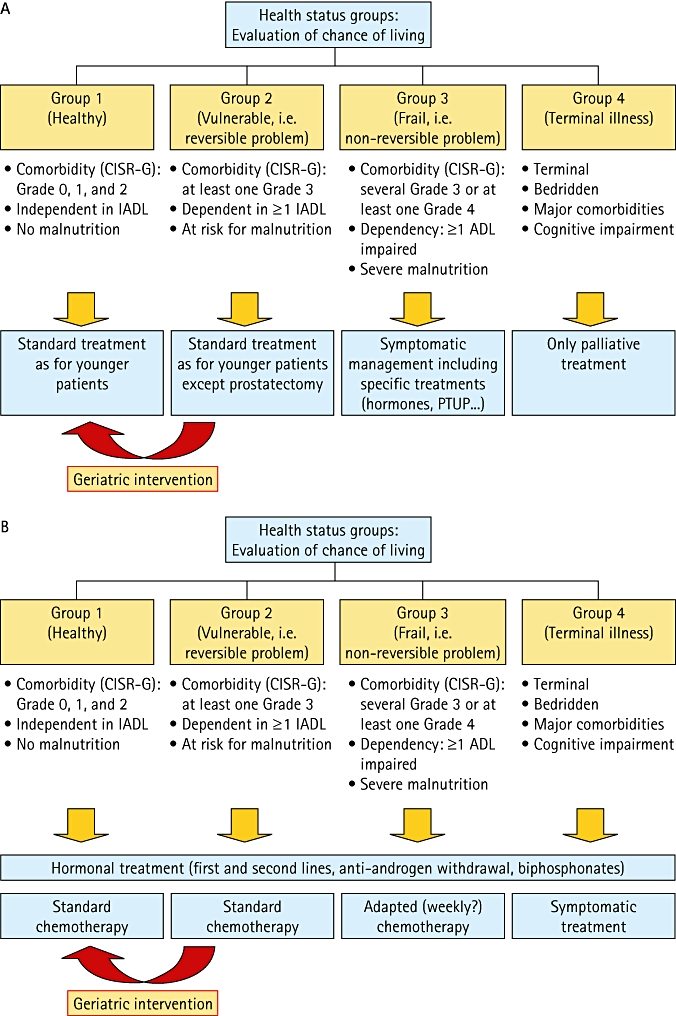
A decision tree for treating patients with: **A**, localized disease; and **B**, metastatic disease.

These groups, rather than chronological age, should be used when considering treatment options for localized and advanced prostate cancer (Fig. 2A,B).

## TREATMENT OF PROSTATE CANCER

The SIOG Prostate Cancer Working Group examined the standard approaches for managing and treating localized and advanced prostate cancer and applied, when possible, evidence-based considerations specific to the older population.

### Localized prostate cancer

The aim of treatment for localized prostate cancer (T1–3, N0, M0 disease) is generally curative. Older men are more likely to develop larger tumours of a higher grade than are younger patients [18,19]. Nevertheless, there is evidence both from the USA [20] and Europe [21] that only a minority of patients will receive curative treatment. Decisions for treatment in older men with localized prostate cancer should take into consideration the risk of dying from prostate cancer (which depends of the grade and stage of the tumour), the risk of dying from another cause (which depends more on the severity of patient comorbidities than chronological age), potential adverse effects of treatment, and patient preferences.

**FIG. 3:**
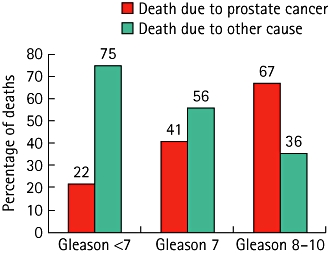
The causes of death in 330 men with clinically localized prostate cancer diagnosed when aged 70–74 years and managed by either surveillance or hormonal therapy for a median of 24 years; from [22].

In a retrospective analysis of 330 men with clinically localized prostate cancer diagnosed at age 70–74 years and managed by either surveillance or hormonal therapy for a median of 24 years, prostate cancer mortality rate was strongly related to the Gleason score, ranging from 22% for patients with a Gleason score of <7 to 67% for those with a Gleason score of ≥8 (Fig. 3) [22].

Several risk-stratification tools and nomograms have been developed to predict pathological stage and outcomes after various treatments. A commonly used tool is that developed by D’Amico *et al*. [23] to evaluate the probability of biochemical relapse at 5 years after curative therapy. Patients are classified into three risk groups (low, medium, and high) based on biopsy Gleason score, preoperative PSA level and clinical stage. According to this classification, there is evidence that older men at high risk of PSA relapse have a significant probability of dying from prostate cancer compared with low- and intermediate-risk patients, and should therefore benefit from curative treatment [24].

### RP

The main recommendations for RP are summarized in Table 1; RP has been shown to improve life-expectancy in older patients with few comorbidities and moderately or poorly differentiated disease [25]. In patients with severe comorbidities, the risk of death from prostate cancer should be carefully balanced with the risk of dying from another cause. The risk of short-term postoperative complications also appears to be more related to the severity of comorbidities than chronological age [26]. Conversely, the risk of long-term incontinence, a common complication after RP, seems markedly more influenced by increasing age than comorbidity [26].

**TABLE 1:** A summary of the guidelines for RP and RT, highlighting references to age. Reprinted from [7], with permission from Elsevier

Guideline, year	Guideline/recommendation
**RP**	
AUA, 2007 update [6]	
	Based on the Expert Panel’s interpretation of the literature and opinion, the patient most likely to benefit from RP would have a relatively long life-expectancy, no significant surgical risk factors, and a preference for surgery
	Candidates for surgery should have: (1) A life-expectancy longer than the expected morbidity of the cancer if left untreated; (2) No significant surgical risk factors or serious comorbid conditions that would contraindicate an elective operation; (3) A willingness to undergo surgery after discussing the risks, operative side-effects, natural history, and options
EAU, 2008 update [4]	
	RP is a standard treatment in patients with stage T1b–T2b, Nx–N0, M0 disease, and a life-expectancy of >10 years
	RP is optional in younger patients with stage T1a disease and a long life-expectancy
	RP is optional for selected patients with limited ≤T3a, Gleason score ≤8, PSA level of <20 ng/mL, and long life-expectancy
NCCN 2009 [5]	
	RP is appropriate for any patient whose tumour is clinically confined to the prostate, has a life-expectancy of ≥10 years and has no serious comorbid conditions that would contraindicate an elective operation
**RT**	
AUA, 2007 update [6]	
	The patient most likely to benefit from RT would have a relatively long life-expectancy, no significant risk factors for radiation toxicity and a preference for RT
	Comment: Insufficient follow-up to compare survival outcomes of EBRT and brachytherapy
NCCN 2009 [5]	
	Treatment recommendations are based on anticipated life-expectancies and risk of recurrence:1 Low risk of recurrence (stage T1–T2a, low Gleason score 2–6, and PSA level <10 ng/mL: RT (3-D EBRT or brachytherapy) is an acceptable strategy in patients whose age or comorbidity leads to a life-expectancy of <10 years and in patients with a life-expectancy of ≥10 years2 Intermediate risk of recurrence (stage T2b–T2c, Gleason score 7, or PSA level 10–20 ng/mL): RT (EBRT with or without brachytherapy) is a treatment option in men with a life-expectancy of <10 or ≥10 years
EAU, 2008 update [4]	
	Treatment decision should be based on TNM classification, Gleason score, baseline PSA level, age, comorbidity, life-expectancy, and HRQL:1 3D-CRT with or without IMRT is recommended for patients with T1c–T2c N0 m0 disease. There is fairly strong evidence that intermediate-risk patients (T2b, PSA 10–20 ng/mL, or Gleason score of 7) benefit from dose escalation.2 Transperineal interstitial brachytherapy without permanent implants can be proposed for patients with cT1–T2a–b, Gleason score <7 (or 3 + 4), PSA ≤10 ng/mL, prostate volume ≤60 mL, without previous TURP, and with a goodIPSS

3D-CRT, three-dimensional conformal RT; IMRT, intensity-modulated RT.

### External beam radiotherapy (EBRT)

The main recommendations in prostate cancer guidelines for EBRT are also summarized in Table 1. Studies have shown similar outcomes in terms of cancer control and treatment-related comorbidities in both older and younger patients. The combination of RT with adjuvant androgen-deprivation therapy (ADT) has been shown to improve the 5- and 8-year survival rates in patients with stage T3/T4 disease, whereas there was no survival advantage for men with T1/T2 disease [27]. However, a recent report by D’Amico *et al*. [28] suggests that this survival benefit might only apply to high-risk patients with no or minimal comorbidities.

### Brachytherapy

Brachytherapy is indicated in patients with low-risk prostate cancer, a prostate volume of <50 cm^3^, and a good IPSS [4]. Brachytherapy might be a suitable option in older patients, but the survival benefit in older men with low-risk disease has not yet been established. Although complications are generally less severe than with RP, urinary, bowel and erectile complications increase significantly with both increasing age and severity of comorbidities [4].

### ADT

In patients with nonmetastatic prostate cancer unsuitable for curative treatment, immediate ADT had a modest benefit on overall survival, but had no effect on prostate cancer mortality and symptom-free survival rates, except possibly in patients with a PSA level of >50 ng/mL and a PSA doubling time of <12 months [29,30]. However, ADT can be used for patients who need symptom palliation and who are unfit for curative therapy.

Besides the lack of compelling evidence for the use of ADT in localized prostate cancer, care is particularly needed in older men, as ADT is associated with an increased risk of fractures [31], diabetes [32] and cardiovascular morbidity [33]. Older men with a low baseline bone mineral density or a high rate of bone loss during hormone therapy could be considered candidates for bisphosphonate therapy.

### ‘Watch-and-wait’ policy and active surveillance

Patients likely to benefit from a ‘watch-and-wait’ policy (i.e. expectant management) or ‘active surveillance’ (involving delayed intervention on progression) are those in the low-risk group as defined by D’Amico *et al*. [24], those who have a short life-expectancy (advanced age, severe comorbidities), and those who express a personal preference to avoid or delay the side-effects of definitive therapy.

For patients in the intermediate-risk group, the risk of dying from prostate cancer should be carefully balanced with the risk of dying from another cause. This group of patients would deserve further evaluation, especially considering that the likelihood of being upstaged from clinical T1–T2 to pathological T3–T4, or upgraded from a biopsy Gleason score of <7 to a pathological Gleason score of 7–10 is higher in men aged ≥70 years than in younger patients [18].

In summary, the decision making is based on the evaluation of the competition between the risk of dying from prostate cancer and the risk of dying from health status impairment. One approach of both aspects, health status being limited to comorbidities evaluated by the Charlson Index, is the practical use of a specific nomogram [34]. Another approach is a combination of a simplified evaluation of health status and a simple evaluation of risk factors for prostate cancer that we have introduced in these recommendations. However, even the choice of a risk factor classification for prostate cancer can be discussed; it was shown that in classification of D’Amico *et al*., the intermediate- and high-risk groups overlapped when using a nomogram-based risk group assessment in patients who were treated by RP [35]. This in favour of considering both risk groups for active treatment even in vulnerable patients. We have chosen the D’Amico *et al*. classification because it is widely used for RT, which is the most frequently used curative treatment in older men.

## EXPERT PANEL RECOMMENDATIONS FOR LOCALIZED PROSTATE CANCER

Treatment decisions should be based on a health status evaluation (mainly driven by the severity of associated comorbidities) rather than chronological age, and on patient preference.

‘Fit’ and ‘vulnerable’ older men in the high-risk group defined by D’Amico *et al*. [23], with a chance of surviving for >10 years are likely to benefit from curative treatment.

Older men in the ‘low-risk’ and possibly in the ‘intermediate-risk’ groups of the risk classification [23] are likely to benefit from an active surveillance approach.

The benefits and harms of ADT for localized prostate cancer should be carefully balanced in older men. Attention is drawn to an increased risk of diabetes, cardiovascular complications, and osteoporosis and bone fractures.

## ADVANCED PROSTATE CANCER

ADT is the mainstay of treatment for patients with metastatic prostate cancer. Surgical castration and castration by LHRH agonists are the standard of care for first-line treatment. There is no established difference in efficacy between these treatments; however, the use of LHRH agonists is usually preferred because it avoids the physical and psychological discomfort of bilateral orchidectomy [4].

The standard procedure for second-line hormonal treatment is cessation of antiandrogen if given as first-line treatment in association with an LHRH agonist. Importantly, there is currently no established survival benefit with second-line and subsequent lines of hormone therapy. When prostate cancer becomes castration-refractory it is recommended that LHRH agonist therapy is continued, but there are no available data specifically supporting this approach in older men.

Given the increased risk of osteoporosis and fracture in older men on ADT, care is needed [5]. All men receiving ADT should receive calcium and vitamin D supplementation, and baseline bone mineral density should be determined. The routine use of bisphosphonates to prevent skeletal complications in patients undergoing ADT is not recommended unless there is a documented risk for fracture or castration-resistant prostate cancer (CRPC) with skeletal metastases.

### Chemotherapy in CRPC

There is growing acceptance that older age, *per se*, is not a contraindication to chemotherapy in older men and that many older patients tolerate it as well as younger patients [5]. Docetaxel-based regimens are the standard of care for patients with CRPC because they provide a survival advantage while reducing pain and improving HRQL [36–38]. In a subgroup analysis, the survival benefit of 3-weekly docetaxel compared with mitoxantrone was consistent between age groups, with hazard ratios for overall survival in patients aged ≤68 and >68 years of 0.81 and 0.77, respectively [36]. Using a more extreme age threshold of >75 years, the hazard ratio was 0.80.

In a retrospective analysis of 175 patients aged ≥75 years treated with docetaxel (either a 3-weekly or a weekly regimen, according to clinical judgement), patients with a good performance status responded to docetaxel therapy to a similar extent as younger patients. Docetaxel was generally well tolerated; the weekly regimen showed less febrile neutropenia than the 3-weekly regimen but a higher rate of fatigue, resulting in frequent treatment discontinuation [39]. In real-life practice, healthy or vulnerable older patients usually receive the 3-weekly regimen, whereas frail patients are more likely to receive the weekly regimen [40].

To date, there is no evidence to support primary prophylaxis with granulocyte colony-stimulating factor in this setting; nevertheless, this agent can be given in selected cases, based on the specific risk of toxicity.

### RT/radiopharmaceuticals

RT is the first choice for localized painful metastasis and is useful in the treatment of painful lesions in older patients with CRPC. As yet, no study has been conducted specifically in older men, but the toxicity profile of radiopharmaceuticals appears appropriate for administration in these patients.

## EXPERT PANEL RECOMMENDATIONS FOR ADVANCED PROSTATE CANCER

- ADT is the first-line treatment in hormone-sensitive metastatic prostate cancer. Evaluation of bone mineral status and prevention of osteoporosis are recommended.
- In metastatic CRPC, chemotherapy with docetaxel (75 mg/m^2^ every 3 weeks) is the standard for fit and vulnerable older men.
- The tolerability of the docetaxel 3-weekly regimen has not been specifically studied in frail older men. The place of weekly docetaxel in metastatic CRPC should be further evaluated.
- Palliative treatments include palliative surgery, radiopharmaceuticals, RT and medical treatments for pain and symptoms.

## PROSTATE CANCER SCREENING

Screening in older men with prostate cancer is highly controversial. Individualized screening decisions should be based on patient health status but not on chronological age.

## SUMMARY

Prostate cancer is a disease of older men; the median age at diagnosis is 68 years, and 75% of deaths due to prostate cancer occur in men aged ≥75 years. There is also evidence that older men are more likely to develop larger tumours of a higher grade than are younger patients. With the exponential ageing of the population and the increasing life-expectancy in developed countries, the burden of prostate cancer is expected to increase dramatically.

The SIOG Prostate Cancer Working Group has developed evidence-based recommendations for the management of older men with prostate cancer which can be summarized as follows:

- The urological approach in older men with prostate cancer should be the same as in younger patients, based on existing international recommendations [4–6].
- Older men with prostate cancer should be managed according to their individual health status, which is mainly driven by the severity of associated comorbid conditions, dependence and nutritional status and not according to chronological age.
- Screening for health status should include evaluation of comorbid conditions (CISR-G scale), dependence status (IADL and ADL scales), and nutritional status (weight loss estimation). In cases of vulnerability and frailty, additional geriatric interventions, including a CGA, might be needed. This allows patients to be easily and rapidly classified into one of four groups:

1. ‘Fit’ or ‘healthy’ older men should receive the same standard treatment as younger patients. More specifically, they should receive curative therapy in cases of high-risk localized prostate cancer and standard chemotherapy in cases of CRPC.
2. ‘Vulnerable’ patients (i.e. reversible impairment) should receive standard treatment after resolution of any geriatric problems through geriatric interventions.
3. ‘Frail’ patients (i.e. irreversible impairment) should receive an adapted treatment.
4. Patients who are ‘too sick’ with ‘terminal illness’ should receive only symptomatic palliative treatment.

## Abbreviations

HRQL: health-related quality of life
SIOG: International Society of Geriatric Oncology
RP: radical prostatectomy
EAU: European Association of Urology
NCCN: National Comprehensive Cancer Network
IADL: Instrumental Activities of Daily Living
ADL: Activities of Daily Living
CGA: comprehensive geriatric assessment
CISR-G: Cumulative Illness Score Rating-Geriatrics
(EB)RT: (external beam) radiotherapy
ADT: androgen-deprivation therapy
CRPC: castration-resistant prostate cancer.
